# (*E*)-2-[(4-Chloro­phen­yl)imino­meth­yl]-4-(trifluoro­meth­oxy)phenol

**DOI:** 10.1107/S1600536809040690

**Published:** 2009-10-10

**Authors:** Marife Tüfekçi, Yelda Bingöl Alpaslan, Mustafa Macit, Ahmet Erdönmez

**Affiliations:** a Department of Physics, Faculty of Arts & Science, Ondokuz Mayıs University, TR-55139 Kurupelit-Samsun, Turkey; b Department of Chemistry, Faculty of Arts & Science, Ondokuz Mayıs University, 55139 Samsun, Turkey

## Abstract

The title compound, C_14_H_9_ClF_3_NO_2_, crystallizes in a phenol–imine tautomeric form, with a strong intra­molecular O—H⋯N hydrogen bond. The dihedral angle between the two benzene rings is 47.62 (9)°. In the crystal, mol­ecules are linked into chains along the *c* axis by C—H⋯O hydrogen bonds, and weak C—H⋯π inter­actions involving both benzene rings are also observed.

## Related literature

For general background to Schiff bases, see: Calligaris *et al.* (1972 ▶); Cohen *et al.* (1964 ▶); Hadjoudis *et al.* (1987 ▶); Karadayı *et al.* (2003 ▶); Hökelek *et al.*(2000 ▶); Dey *et al.* (2001 ▶); Ünver *et al.* (2002 ▶). For a related structure, see: Gül *et al.* (2007 ▶). For hydrogen-bond motifs, see: Bernstein *et al.* (1995 ▶).
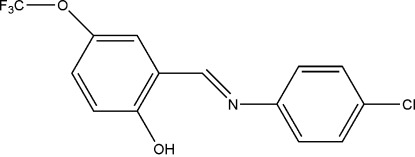


## Experimental

### 

#### Crystal data

C_14_H_9_ClF_3_NO_2_
*M* *_r_* = 315.67
Monoclinic, 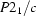

*a* = 29.612 (5) Å
*b* = 7.195 (5) Å
*c* = 6.375 (5) Å
β = 96.012 (5)°
*V* = 1350.8 (14) Å^3^
*Z* = 4
Mo *K*α radiation
μ = 0.32 mm^−1^
*T* = 296 K
0.72 × 0.44 × 0.10 mm

#### Data collection

Stoe IPDS-II diffractometer
Absorption correction: integration (*X-RED32*; Stoe & Cie, 2002 ▶) *T*_min_ = 0.844, *T*_max_ = 0.966
11226 measured reflections
2579 independent reflections
1539 reflections with *I* > 2σ(*I*)
*R*_int_ = 0.059

#### Refinement

*R*[*F* ^2^ > 2σ(*F* ^2^)] = 0.062
*wR*(*F* ^2^) = 0.201
*S* = 1.04
2579 reflections
190 parameters
H-atom parameters constrained
Δρ_max_ = 0.32 e Å^−3^
Δρ_min_ = −0.33 e Å^−3^

### 

Data collection: *X-AREA* (Stoe & Cie, 2002 ▶); cell refinement: *X-AREA*; data reduction: *X-RED32* (Stoe & Cie, 2002 ▶); program(s) used to solve structure: *SHELXS97* (Sheldrick, 2008 ▶); program(s) used to refine structure: *SHELXL97* (Sheldrick, 2008 ▶); molecular graphics: *ORTEP-3 for Windows* (Farrugia, 1997 ▶); software used to prepare material for publication: *WinGX* (Farrugia, 1999 ▶).

## Supplementary Material

Crystal structure: contains datablocks I, global. DOI: 10.1107/S1600536809040690/ci2934sup1.cif

Structure factors: contains datablocks I. DOI: 10.1107/S1600536809040690/ci2934Isup2.hkl

Additional supplementary materials:  crystallographic information; 3D view; checkCIF report

## Figures and Tables

**Table 1:** Hydrogen-bond geometry (Å, °)

*D*—H⋯*A*	*D*—H	H⋯*A*	*D*⋯*A*	*D*—H⋯*A*
O1—H1⋯N1	0.82	1.88	2.604 (4)	147
C14—H14⋯O1^i^	0.93	2.53	3.396 (5)	155
C2—H2⋯*Cg*1^ii^	0.93	2.77	3.496 (4)	135
C5—H5⋯*Cg*1^iii^	0.93	2.98	3.713 (4)	136
C10—H10⋯*Cg*2^iv^	0.93	2.94	3.644 (4)	133
C13—H13⋯*Cg*2^v^	0.93	2.88	3.597 (4)	135

Symmetry codes: (i) 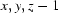; (ii) 
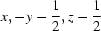
; (iii) 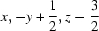; (iv) 
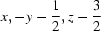
; (v) 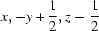. *Cg*1 and *Cg*2 are the centroids of the C1–C6 and C9–C14 rings, respectively.

**Fig. 1.**
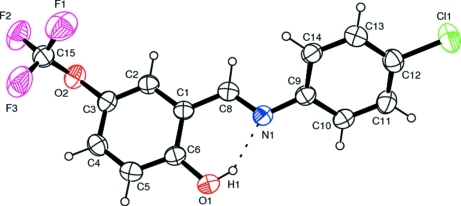
The molecular structure of (I), showing the atom-numbering scheme. Displacement ellipsoids are drawn at the 30% probability level and H atoms are shown as small spheres of arbitrary radii. The dashed line indicates a hydrogen bond.

**Fig. 2.**
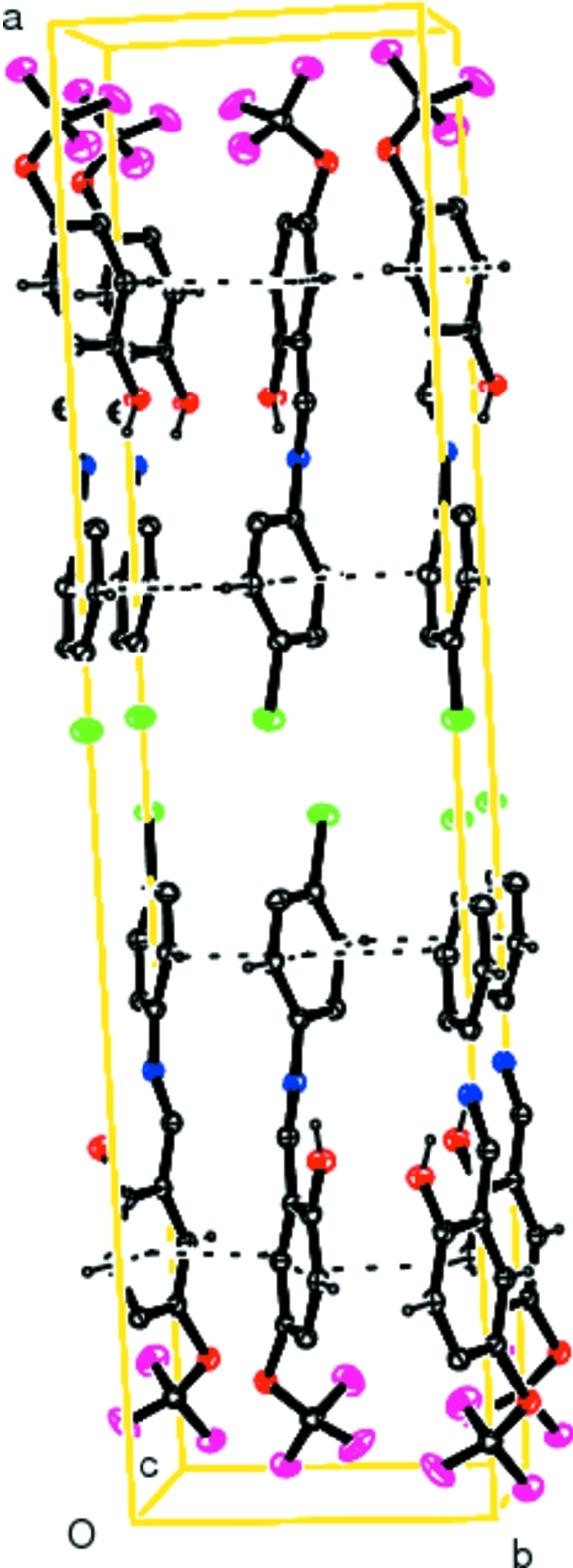
A packing diagram for (I). H atoms not involved in hydrogen bonding (dashed lines) have been omitted for clarity.

## supplementary crystallographic information

### Comment

Schiff bases have been extensively used as ligands in the field of coordination chemistry (Calligaris *et al.*, 1972). Schiff base compounds can be classified by their photochromic and thermochromic characteristics (Cohen *et al.*, 1964). These properties result from a proton transfer from the hydroxyl O atom to the imine N atom (Hadjoudis *et al.*,  1987). Schiff bases display two possible tautomeric forms, namely the phenol-imine (Dey *et al.*,  2001; Karadayı *et al.*, 2003) and keto-amine  (Hökelek *et al.*,  2000) forms. Our X-ray analysis shows that the title compound, (I), exists in the phenol-imine form (Fig. 1).

The C8═N1 [1.282 (4) Å] and C6—O1 [1.343 (4) Å] bond lenghts confirm the phenol-imine form of (I), and these distances are similar to those reported in the literature  [1.280 (2) Å and 1.350 (3) Å; Gül *et al.*,  2007]. The molecule is not planar and the dihedral angle between the C1-C6 and C9-C14 rings is 47.62 (9)°. A strong intramolecular O1—H1···N1 hydrogen bond which forms an S(6) graph set motif (Bernstein *et al.*,  1995) is observed.

The crystal packing is stabilized by intermolecular C—H···O hydrogen bonds (Table 1). In addition, C2—H2···Cg1^i^, C5—H5···Cg1^ii^, C10—H10···Cg2^iii^ and C13—H13···Cg2^iv^ (Cg1 and Cg2 are centroids of the C1-C6 and C9-C14 rings, respectively) interactions (Fig.2 and Table 1) are observed.

### Experimental

2-[(4-Chlorophenyl)iminomethyl]-4-trifluoromethoxyphenol was prepared by refluxing a mixture of a solution containing 2-hydroxy-5-(trifluoromethoxy)benzaldehyde (10 mg, 4.85× 10^-2^ mmol) in ethanol (20 ml) and a solution containing 4-chloroaniline (10 mg, 4.85× 10^-2^ mmol) in ethanol (20 ml). The reaction mixture was stirred for 1 hour under reflux. Single crystals of the title compound for X-ray analysis were obtained by slow evaporation of an ethanol solution (yield 44 %; m.p. 376-377 K).

### Refinement

All H atoms were placed in calculated positions and constrained to ride on their parent atoms, with O-H = 0.82 Å, C-H = 0.93 Å and *U*_iso_(H) = 1.2*U*_eq_(C).

### Crystal data

**Table:**

C_14_H_9_ClF_3_NO_2_	*F*(000) = 640
*M**_r_* = 315.67	*D*_x_ = 1.552 Mg m^−^^3^
Monoclinic, *P*2_1_/*c*	Mo *K*α radiation, λ = 0.71069 Å
Hall symbol: -P 2ybc	Cell parameters from 13303 reflections
*a* = 29.612 (5) Å	θ = 2.1–26.7°
*b* = 7.195 (5) Å	µ = 0.32 mm^−^^1^
*c* = 6.375 (5) Å	*T* = 296 K
β = 96.012 (5)°	Plate, light brown
*V* = 1350.8 (14) Å^3^	0.72 × 0.44 × 0.10 mm
*Z* = 4	

### Data collection

**Table:**

Stoe IPDS-II diffractometer	2579 independent reflections
Radiation source: fine-focus sealed tube	1539 reflections with *I* > 2σ(*I*)
graphite	*R*_int_ = 0.059
Detector resolution: 6.67 pixels mm^-1^	θ_max_ = 26.0°, θ_min_ = 2.8°
ω scans	*h* = −36→32
Absorption correction: integration (*X-RED32*; Stoe & Cie, 2002)	*k* = −8→8
*T*_min_ = 0.844, *T*_max_ = 0.966	*l* = −7→7
11226 measured reflections	

### Refinement

**Table:**

Refinement on *F*^2^	Primary atom site location: structure-invariant direct methods
Least-squares matrix: full	Secondary atom site location: difference Fourier map
*R*[*F*^2^ > 2σ(*F*^2^)] = 0.062	Hydrogen site location: inferred from neighbouring sites
*wR*(*F*^2^) = 0.201	H-atom parameters constrained
*S* = 1.04	*w* = 1/[σ^2^(*F*_o_^2^) + (0.1139*P*)^2^] where *P* = (*F*_o_^2^ + 2*F*_c_^2^)/3
2579 reflections	(Δ/σ)_max_ = 0.001
190 parameters	Δρ_max_ = 0.32 e Å^−^^3^
0 restraints	Δρ_min_ = −0.33 e Å^−^^3^

### Special details

**Table:**

Geometry. All esds (except the esd in the dihedral angle between two l.s. planes) are estimated using the full covariance matrix. The cell esds are taken into account individually in the estimation of esds in distances, angles and torsion angles; correlations between esds in cell parameters are only used when they are defined by crystal symmetry. An approximate (isotropic) treatment of cell esds is used for estimating esds involving l.s. planes.
Refinement. Refinement of F^2^ against ALL reflections. The weighted R-factor wR and goodness of fit S are based on F^2^, conventional R-factors R are based on F, with F set to zero for negative F^2^. The threshold expression of F^2^ > 2sigma(F^2^) is used only for calculating R-factors(gt) etc. and is not relevant to the choice of reflections for refinement. R-factors based on F^2^ are statistically about twice as large as those based on F, and R- factors based on ALL data will be even larger.

### Fractional atomic coordinates and isotropic or equivalent isotropic displacement parameters (Å^2^)

**Table:**

	*x*	*y*	*z*	*U*_iso_*/*U*_eq_	
C1	0.79069 (11)	0.5332 (4)	0.8304 (4)	0.0561 (7)	
C2	0.83127 (11)	0.5846 (4)	0.7543 (5)	0.0595 (8)	
H2	0.8309	0.6345	0.6196	0.071*	
C3	0.87134 (11)	0.5624 (4)	0.8759 (5)	0.0612 (8)	
C4	0.87339 (12)	0.4841 (4)	1.0761 (5)	0.0651 (8)	
H4	0.9012	0.4679	1.1566	0.078*	
C5	0.83396 (12)	0.4317 (4)	1.1521 (5)	0.0662 (9)	
H5	0.8351	0.3769	1.2847	0.079*	
C6	0.79214 (11)	0.4581 (4)	1.0363 (5)	0.0607 (8)	
C8	0.74786 (12)	0.5520 (4)	0.6986 (5)	0.0616 (8)	
H8	0.7481	0.5908	0.5596	0.074*	
C9	0.66929 (11)	0.5123 (4)	0.6311 (5)	0.0598 (7)	
C10	0.62906 (12)	0.5708 (4)	0.7062 (5)	0.0674 (8)	
H10	0.6298	0.6195	0.8417	0.081*	
C11	0.58855 (13)	0.5576 (5)	0.5832 (6)	0.0778 (10)	
H11	0.5619	0.5972	0.6350	0.093*	
C12	0.58717 (12)	0.4860 (5)	0.3835 (6)	0.0716 (9)	
C13	0.62616 (13)	0.4247 (5)	0.3058 (5)	0.0707 (9)	
H13	0.6248	0.3734	0.1713	0.085*	
C14	0.66709 (12)	0.4392 (4)	0.4268 (5)	0.0637 (8)	
H14	0.6935	0.4003	0.3729	0.076*	
C15	0.94016 (13)	0.5208 (5)	0.7263 (6)	0.0748 (9)	
N1	0.70971 (9)	0.5168 (3)	0.7683 (4)	0.0618 (7)	
O1	0.75423 (9)	0.4102 (3)	1.1218 (4)	0.0773 (7)	
H1	0.7320	0.4327	1.0380	0.116*	
O2	0.91140 (8)	0.6360 (3)	0.8050 (4)	0.0756 (7)	
F1	0.91975 (12)	0.4373 (4)	0.5572 (5)	0.1403 (12)	
F2	0.97374 (9)	0.6120 (4)	0.6655 (5)	0.1174 (9)	
F3	0.95437 (12)	0.3876 (5)	0.8491 (6)	0.1558 (15)	
Cl1	0.53598 (4)	0.46496 (19)	0.22578 (19)	0.1103 (5)	

### Atomic displacement parameters (Å^2^)

**Table:**

	*U*^11^	*U*^22^	*U*^33^	*U*^12^	*U*^13^	*U*^23^
C1	0.0635 (19)	0.0520 (15)	0.0530 (16)	−0.0008 (13)	0.0076 (13)	−0.0016 (12)
C2	0.068 (2)	0.0537 (15)	0.0575 (16)	−0.0002 (13)	0.0091 (15)	0.0024 (13)
C3	0.0584 (19)	0.0548 (16)	0.0713 (19)	−0.0048 (13)	0.0118 (15)	−0.0032 (13)
C4	0.067 (2)	0.0586 (17)	0.0674 (18)	0.0012 (14)	−0.0043 (15)	−0.0038 (14)
C5	0.081 (3)	0.0627 (18)	0.0551 (17)	0.0009 (15)	0.0063 (16)	−0.0006 (13)
C6	0.067 (2)	0.0583 (17)	0.0576 (17)	−0.0009 (14)	0.0121 (15)	−0.0030 (13)
C8	0.069 (2)	0.0602 (17)	0.0557 (16)	−0.0005 (14)	0.0073 (15)	0.0022 (13)
C9	0.063 (2)	0.0565 (15)	0.0609 (17)	−0.0012 (13)	0.0101 (15)	0.0027 (13)
C10	0.067 (2)	0.076 (2)	0.0608 (18)	0.0042 (16)	0.0156 (16)	−0.0032 (15)
C11	0.059 (2)	0.089 (2)	0.087 (2)	0.0072 (17)	0.0131 (18)	0.0024 (19)
C12	0.066 (2)	0.0692 (19)	0.077 (2)	−0.0015 (16)	−0.0024 (17)	0.0100 (17)
C13	0.076 (3)	0.074 (2)	0.0614 (18)	0.0006 (16)	0.0042 (17)	0.0013 (15)
C14	0.062 (2)	0.0690 (19)	0.0614 (18)	0.0028 (14)	0.0144 (15)	0.0015 (14)
C15	0.070 (2)	0.079 (2)	0.075 (2)	−0.0030 (19)	0.0089 (18)	0.0058 (19)
N1	0.0598 (17)	0.0655 (15)	0.0608 (15)	0.0022 (12)	0.0094 (13)	0.0018 (11)
O1	0.0702 (16)	0.1015 (17)	0.0622 (13)	−0.0015 (12)	0.0164 (11)	0.0150 (12)
O2	0.0702 (16)	0.0633 (13)	0.0952 (16)	−0.0058 (11)	0.0180 (13)	−0.0014 (11)
F1	0.137 (3)	0.158 (3)	0.130 (2)	−0.025 (2)	0.032 (2)	−0.063 (2)
F2	0.0774 (17)	0.126 (2)	0.154 (2)	−0.0122 (14)	0.0383 (16)	0.0144 (17)
F3	0.129 (3)	0.169 (3)	0.181 (3)	0.081 (2)	0.066 (2)	0.091 (2)
Cl1	0.0737 (8)	0.1402 (10)	0.1111 (9)	−0.0048 (6)	−0.0188 (6)	0.0037 (7)

### Geometric parameters (Å, °)

**Table:**

C1—C2	1.392 (4)	C9—N1	1.407 (4)
C1—C6	1.416 (4)	C10—C11	1.366 (5)
C1—C8	1.453 (4)	C10—H10	0.93
C2—C3	1.357 (5)	C11—C12	1.370 (5)
C2—H2	0.93	C11—H11	0.93
C3—C4	1.391 (5)	C12—C13	1.376 (5)
C3—O2	1.416 (4)	C12—Cl1	1.736 (4)
C4—C5	1.363 (5)	C13—C14	1.371 (5)
C4—H4	0.93	C13—H13	0.93
C5—C6	1.387 (5)	C14—H14	0.93
C5—H5	0.93	C15—F3	1.280 (4)
C6—O1	1.343 (4)	C15—F2	1.285 (4)
C8—N1	1.282 (4)	C15—F1	1.323 (5)
C8—H8	0.93	C15—O2	1.324 (4)
C9—C10	1.394 (5)	O1—H1	0.82
C9—C14	1.400 (4)		
			
C2—C1—C6	118.7 (3)	C11—C10—C9	120.8 (3)
C2—C1—C8	120.4 (3)	C11—C10—H10	119.6
C6—C1—C8	120.8 (3)	C9—C10—H10	119.6
C3—C2—C1	120.3 (3)	C10—C11—C12	119.9 (3)
C3—C2—H2	119.8	C10—C11—H11	120.1
C1—C2—H2	119.8	C12—C11—H11	120.1
C2—C3—C4	121.6 (3)	C11—C12—C13	120.7 (3)
C2—C3—O2	119.1 (3)	C11—C12—Cl1	120.7 (3)
C4—C3—O2	119.1 (3)	C13—C12—Cl1	118.6 (3)
C5—C4—C3	118.8 (3)	C14—C13—C12	119.9 (3)
C5—C4—H4	120.6	C14—C13—H13	120.0
C3—C4—H4	120.6	C12—C13—H13	120.0
C4—C5—C6	121.5 (3)	C13—C14—C9	120.3 (3)
C4—C5—H5	119.2	C13—C14—H14	119.8
C6—C5—H5	119.2	C9—C14—H14	119.8
O1—C6—C5	119.1 (3)	F3—C15—F2	110.6 (4)
O1—C6—C1	121.9 (3)	F3—C15—F1	104.5 (4)
C5—C6—C1	119.0 (3)	F2—C15—F1	106.8 (3)
N1—C8—C1	121.9 (3)	F3—C15—O2	114.8 (3)
N1—C8—H8	119.0	F2—C15—O2	110.1 (3)
C1—C8—H8	119.0	F1—C15—O2	109.6 (3)
C10—C9—C14	118.3 (3)	C8—N1—C9	120.8 (3)
C10—C9—N1	118.8 (3)	C6—O1—H1	109.5
C14—C9—N1	122.7 (3)	C15—O2—C3	118.7 (3)
			
C6—C1—C2—C3	0.1 (4)	C9—C10—C11—C12	0.0 (5)
C8—C1—C2—C3	−178.3 (3)	C10—C11—C12—C13	0.8 (5)
C1—C2—C3—C4	1.7 (4)	C10—C11—C12—Cl1	179.1 (3)
C1—C2—C3—O2	−172.5 (2)	C11—C12—C13—C14	−1.6 (5)
C2—C3—C4—C5	−1.1 (5)	Cl1—C12—C13—C14	−179.9 (2)
O2—C3—C4—C5	173.1 (3)	C12—C13—C14—C9	1.5 (5)
C3—C4—C5—C6	−1.3 (5)	C10—C9—C14—C13	−0.6 (4)
C4—C5—C6—O1	−177.5 (3)	N1—C9—C14—C13	174.8 (3)
C4—C5—C6—C1	3.0 (4)	C1—C8—N1—C9	−171.6 (3)
C2—C1—C6—O1	178.2 (3)	C10—C9—N1—C8	−146.5 (3)
C8—C1—C6—O1	−3.4 (4)	C14—C9—N1—C8	38.1 (4)
C2—C1—C6—C5	−2.4 (4)	F3—C15—O2—C3	−56.1 (5)
C8—C1—C6—C5	176.0 (3)	F2—C15—O2—C3	178.3 (3)
C2—C1—C8—N1	−175.6 (3)	F1—C15—O2—C3	61.2 (4)
C6—C1—C8—N1	6.0 (4)	C2—C3—O2—C15	−103.8 (4)
C14—C9—C10—C11	−0.2 (5)	C4—C3—O2—C15	81.9 (4)
N1—C9—C10—C11	−175.7 (3)		

### Hydrogen-bond geometry (Å, °)

**Table:**

*D*—H···*A*	*D*—H	H···*A*	*D*···*A*	*D*—H···*A*
O1—H1···N1	0.82	1.88	2.604 (4)	147
C14—H14···O1^i^	0.93	2.53	3.396 (5)	155
C2—H2···Cg1^ii^	0.93	2.77	3.496 (4)	135
C5—H5···Cg1^iii^	0.93	2.98	3.713 (4)	136
C10—H10···Cg2^iv^	0.93	2.94	3.644 (4)	133
C13—H13···Cg2^v^	0.93	2.88	3.597 (4)	135

Symmetry codes: (i) *x*, *y*, *z*−1; (ii) *x*, −*y*−1/2, *z*−1/2; (iii) *x*, −*y*+1/2, *z*−3/2; (iv) *x*, −*y*−1/2, *z*−3/2; (v) *x*, −*y*+1/2, *z*−1/2.
